# Gene expression analysis of porcine whole blood cells infected with foot-and-mouth disease virus using high-throughput sequencing technology

**DOI:** 10.1371/journal.pone.0200081

**Published:** 2018-07-06

**Authors:** Jianliang Lv, Yaozhong Ding, Xinsheng Liu, Li Pan, Zhongwang Zhang, Peng Zhou, Yongguang Zhang, Yonghao Hu

**Affiliations:** 1 College of Veterinary Medicine, Gansu Agricultural University, Lanzhou, Gansu, P. R. China; 2 State Key Laboratory of Veterinary Etiological Biology, National Foot and Mouth Disease Reference Laboratory, Lanzhou Veterinary Research Institute, Chinese Academy of Agricultural Sciences, Lanzhou, Gansu, P. R. China; 3 Jiangsu Co-innovation Center for Prevention and Control of Important Animal Infectious Diseases and Zoonoses, Yangzhou, Jiangsu Province, People's Republic of China; Universitat de Lleida, SPAIN

## Abstract

Foot-and-mouth disease virus (FMDV) is a single-stranded positive RNA virus that belongs to the family *Picornaviridae*. FMDV infects cloven-hoofed animals, such as pigs, sheep, goats, cattle and diverse wildlife species, and remains a major threat to the livestock industry worldwide. In this study, a transcriptome analysis of whole blood from pigs infected with FMDV was performed using the paired-end Illumina sequencing technique to understand the interactions between the pathogen and its host cells. During infection with FMDV, a total of 120 differentially expressed genes (DEGs) were identified, including 110 up-regulated genes and 10 down-regulated genes. To further investigate the DEGs involved in interactions between the virus and its host, gene ontology (GO) annotation and Kyoto Encyclopedia of Genes and Genomes (KEGG) enrichment were conducted. GO annotation indicated that a number of DEGs were enriched in categories involved in host-virus interactions, such as response to stimulus, immune system process and regulation of biological process. KEGG enrichment analysis indicated that the DEGs were primarily involved in the ribosome signaling pathway and immune-related signaling pathways. Ten DEGs, including the immune-related genes *BTK1*, *C1QB*, *TIMD4* and *CXCL10*, were selected and validated using quantitative PCR, which showed that the expression patterns of these genes are consistent with the results of the *in silico* expression analysis. In conclusion, this study presents the first transcriptome analysis of pig whole blood cells infected with FMDV, and the results obtained in this study improve our understanding of the interactions between FMDV and host cells as well as the diagnosis and control of FMD.

## Introduction

Foot-and-mouth disease (FMD) is an acute and highly contagious disease of domesticated and wild cloven-hoofed mammals and is characterized by fever, reduced appetite, and blisters on the snout, tongue, feet and teats that cause salivation and lameness [1]. This disease is caused by the FMD virus (FMDV), which replicates rapidly in the host and spreads to susceptible animals via contact [2]. FMDV does not lead to high mortality in adult animals, but the virus has debilitating effects, such as weight loss, milk drop, loss of draught power and death, in young animals, resulting in severe economic losses and threats to food security [3,4].

The FMDV virion contains a single stranded, plus-sense RNA genome that is approximately 8.4 kb in length. The RNA is translated as a single long open reading frame into a polyprotein; this process is followed by a series of post-translational proteolytic cleavages that produce the intermediate and mature structural and non-structural (NS) viral proteins [5]. The FMDV genome is divided into four main regions based on the initial cleavage products [6]. The L region is located at the 5’ end of the FMDV genome and encodes two L proteins: Lab and Lb [7,8]. Directly downstream of the L region is the P1 region, which encodes four viral structural proteins, namely, VP1, 2, 3 and 4. Following the P1 region is the P2 region, which encodes three viral NS proteins: 2A, 2B and 2C. The P3 region is situated at the 3’ end of the genome and encodes NS proteins, including 3A, three copies of VPg, 3C^pro^ and 3D^pro^ [1]. The FMDV genome is encased by a non-enveloped capsid with icosahedral symmetry that is 28–30 nm in diameter and composed of 60 asymmetrical subunits, each made up of one copy of four structural proteins, VP1 to 4 [3]. FMDVs are grouped into seven immunologically distinct serotypes, namely, O, A, C, Asia 1, South African Territories (SAT) 1, SAT 2, and SAT 3 [1]. The high mutation rate of FMDV leads to rapid evolution and extensive variation between and within serotypes [9].

FMDV can be passed from one animal to another by direct contact or by contact with contaminated objects. The cell tropism of FMDV is largely dependent on the interactions between the G-H loop of capsid VP1 and its cell surface receptor, integrin [10]. Once the FMD virion is captured by its cognate receptors, the virus elicits rapid and broad immune mechanisms [1]. Assessing the immune responses at the gene-expression level may assist in the diagnosis and understanding of the interactions between pathogens and their hosts. Whole blood contains a mixed population of white blood cells, including polymorphonuclear and mononuclear cell populations. Mononuclear cells are further divided into monocytes, macrophages and lymphocytes, which include B cells, T cells and NK cells [11]. Previous studies showed that whole blood, which is an accessible bio-fluid that carries immune cells, is a logical medium in which to study immune response-related gene expression in bacterial and viral infections [12].

High-throughput RNA sequencing techniques are characterized by high sensitivity, genome-wide coverage and unbiased quantification of gene transcription [13]. Transcriptome analysis using RNA-seq is an efficient and powerful method for the identification of immune responsive genes in non-model organisms. In this study, we conducted analyses of the whole blood transcriptome of FMDV-infected and uninfected swine, including transcriptome sequencing, assembly and annotation. This transcriptome dataset described, for the first time, the changes in gene expression in whole blood samples before and after FMDV infection. Moreover, this dataset is a valuable resource for the identification of immune responsive genes and the development of genetic markers for FMD diagnosis in pigs.

## Material and methods

### Animal use and ethics statement

Experimental pigs, bought from the Lintao pig farm in Gansu Province, weighing 30 kg to 35 kg, with no maternal antibodies to FMDV, were maintained under pathogen-free conditions and were fed with pathogen-free food and water. The daily food (mixed feed containing corn, soybean, straw powder and Chinese cabbage) can satisfy the nutrient demand of pigs. The animal house measures 5m×3m×3m and is suitable for one pig (total 12 pigs in 12 rooms), with appropriate temperature, humidity and light. The health of pigs was monitored twice daily after FMDV infection. All pig experiments were performed in a bio-safety level 3 animal facilities of State Key Laboratory of Veterinary Etiological Biology at Lanzhou Veterinary Research Institute (LVRI) following the protocol approved by Gansu Provincial Science and Technology Department and conformed to the local (Regulations for the administration of affairs concerning experimental animals) and international (Dolan K. 2007 Second Edition of Laboratory Animal Law. Blackwell, UK) guidelines on the ethical use of animals. The experiment was also approved by the animal ethics committee of the LVRI, Chinese Academy of Agricultural Sciences (Permission No. SYXK2010-0003). All pigs were humanely euthanized by intravenous injection with 2% pentobarbital sodium (25 mg/Kg) at the end of this study.

### FMDV infection and sampling procedures

Twelve pigs were randomly divided into two groups: non-infected (NI) and experimentally infected (I). After a period of 5 days of acclimatization in the animal rooms, a dose of 10,000 TCID_50_ of the FMDV strain O/GZBY/2010 was administered by muscular injection to stimulate infection, and the NI group was administered an identical volume of sterile phosphate-buffered saline (PBS). Prior to infection, serum and whole blood were collected from each pig to confirm that the animals were negative for antibodies to FMDV and viral RNA using ELISA and RT-PCR. The pigs were also screened for classical swine fever virus (CSFV), porcine circovirus type 2 (PCV2), porcine reproductive and respiratory syndrome virus (PPRSV) and pseudorabies virus (PRV) using serologic tests or in-house PCR. All 6 pigs in the experimental group developed severe clinical disease 3 to 10 days post infection (dpi). They started exhibiting fever and the first vesicles appeared 3 to 4 dpi. Severe vesicular lesions on feet resulted in lameness by 7 to 9 dpi. In contrast, none of the pigs in the PBS group showed clinical signs of FMD during the course of the experiment. Whole blood was collected at 10 dpi. The positive infections were approved by RT-PCR and ELISA (S1 and S2 Figs).

### RNA isolation and RNA sequencing

The whole blood samples from the NI and I groups were collected in PAXgene Blood RNA Tubes (762165). RNA isolation was performed using the RNeasy Protect Animal Blood Kit (Qiagen, 73224), followed by DNase I (NEB, 0303S) treatment at 30°C for 30 min to remove contaminating DNA. An Agilent 2100 Bioanalyzer analysis was conducted on all samples to check the integrity of the total RNA. Individual libraries for sequencing were constructed using the NEBNext Ultra RNA Library Prep Kit for Illumina (NEB, E7430S) according to the manufacturer’s instructions. Paired-end sequencing (100 base pairs) was conducted on an Illumina Hiseq 2000 machine (Novogene Bioinformatics Technology Co., Ltd.).

### Read processing and assembly

The raw reads generated from twelve libraries were subjected to quality control using FastQC [14]. Reads containing adapter or poly-N and low-quality reads (the read quality threshold: the percentage of low quality bases is over 50% in a read, the low quality base is defined as a base whose Q score is no more than 10) were trimmed using FASTQ/A clipper (http://hannonlab.cshl.edu/fastx_toolkit/). Subsequently, clean reads were concatenated and mapped to the pig reference genome (https://www.ncbi.nlm.nih.gov/genome/?term=pig) using TopHat v2.0.9 [15]. Meanwhile, the Q20, Q30 and GC-content of clean reads were calculated (S1 Dataset).

### Transcriptomic and functional annotation analyses

Differentially expressed genes (DEGs) in twelve libraries were identified using the edgeR program package [16]. P-values were adjusted using q-values. A q-value <0.005 & |log2(fold change)|>1 was set as the threshold for significantly different expression [17,18]. The DEGs were then subjected to gene ontology (GO) functional enrichment analysis and Kyoto Encyclopedia of Genes and Genome (KEGG) pathway analysis. GO analysis of the DEGs was implemented by the GOseq R package, which can adjust for gene length bias in DEGs [19]. The KOBAS software was used to test the statistical enrichment of DEGs in KEGG pathways [20].

### qRT-PCR

The cDNA was reversely transcribed from the same RNA templates that were used for sequencing by using the reverse transcription kit (TAKARA, RR037A) according to the manufacturer’s instructions. Specific primers for 10 randomly selected DEGs were designed by the Sigma qPCR primer design online program and listed in Table 1. β-actin was used as an internal reference. The qPCR reactions were carried out in an ABI PRISM 7500 sequence detection system. A quantitative PCR reaction contained 5 μL of the 2× SensiFAST SYBR No-Rox mix (Bioline, BIO-98080), 2–10 ng of cDNA template, and 400 nM of each primer in a total volume of 10 μL. The PCR procedures were as follows: 95°C for 2 min, followed by 40 cycles of 95°C for 5 s, 60°C for 10 s, and 72°C for 10 s. All PCR reactions were conducted in duplicate. The data were presented as the mean ± standard error. Statistical analysis was performed, and p<0.05 was considered to be statistically significant.

**Table 1:** Details of primers used for qPCR assay.

Gene Name	Abbreviation	Primer sequence (5’-3’)
β-actin	**/**	F-AGATCAAGATCATCGCGCCT R-ATGCAACTAACAGTCCGCCT
NADH dehydrogenase subunit 2	NADH2	F- TTCCTAACACAAGCCACAGCCTCCATA R- TGCCTTGGGTTACTTCTGGGACTCA
NADH dehydrogenase subunit 4	NADH4	F-GTCATATAGCACTTGTAATCGTAGCA R-AGGCAGAATAGTATGGAGGATGTTA
ATP Synthase 8	ATP8	F-TGCCACAACTAGATACATCCACATGATTCA R-GATTCTGGGCTTGCTGGGTATGAGTA
Mitochondrial ribosomal protein S23	MRPS23	F-TGCGATACGGCAAAGCCAAAGC R-GTTGACAGGTGGACTTGAAGTTCGGATT
Lin-37 DREAM MuvB core complex component	LIN-37	F-CGATCTGCCGAGCCTGGATG R-CTCTTGCTGTTGGTGACCTCTGAA
Cell division cycle 123	CDC123	F-CTTGAGCGAAGGCGAAGC R-TTGAGAGGTCCACGAAGTCC
T-cell immunoglobulin and mucin domain containing 4	TIMD4	F-TTGTCTGACTCCAACTGCCG R-TTGGCTGACTTCCTCGACAC
Bruton Tyrosine Kinase 1	BTK1	F-TGTGTTCCACACCTCAGAGC R-TTTCCCATGATCCGTAGCCC
Complement C1q B	C1QB	F-ACTTCCGCTTTGGACTGAGAG R-GCTGCTTCCTGGGAACCTGAT
C-X-C motif chemokine 10	CXCL10	F-CCTGCAAGTCAATCTTGCCC R-TCGAGGAGATCTTTTAGACCTTTCT

## Results

### Transcriptome sequencing and assembly

Raw sequence data were submitted to the NCBI short read archive (SRA) portal under accession numbers SAMN07821329-7821340 under the NCBI Bio-project PRJNA415229. The raw reads generated from 12 libraries are summarized in Table 2. Approximately 52 million raw reads were obtained from each library in the NI and I groups. After the removal of reads containing adapter, poly-N and low-quality reads, we obtained approximately 49 million clean reads from each sample. Most of the clean reads are distributed in exon regions, followed by intron regions and intergenic regions. The percentage of clean reads from each library that mapped to the pig reference genome (ftp://ftp.ncbi.nlm.nih.gov/genomes/Sus_scrofa/) was greater than 70%.

**Table 2:** Transcriptome data for the control and FMDV-infected samples.

Group	Sample	SRA accession	Number of raw reads	Number of clean reads	Mapped reads
NI	NI_1	SAMN07821335	51848982	49247102(94.98%)	36259906(73.63%)
	NI_2	SAMN07821336	51849458	49171608(94.84%)	36656568(74.55%)
	NI_3	SAMN07821337	52364394	49861518(95.22%)	37275007(74.76%)
	NI_4	SAMN07821338	51849628	49674698(95.81%)	37407837(75.31%)
	NI_5	SAMN07821339	52364226	49706300(94.92%)	37048670(74.53%)
	NI_6	SAMN07821340	52364530	49815524(95.13%)	36796250(73.87%)
I	I_1	SAMN07821329	52363786	49656494(94.83%)	35714013(71.92%)
	I_2	SAMN07821330	52364320	49815494(95.13%)	36599638(73.47%)
	I_3	SAMN07821331	52364512	49697432(94.91%)	35254833(70.94%)
	I_4	SAMN07821332	52363926	49626430(94.77%)	36193273(72.93%)
	I_5	SAMN07821333	52364226	49706300(94.92%)	37192953(74.83%)
	I_6	SAMN07821334	52363996	49635002(94.79%)	35661983(71.85%)

### Differentially expressed genes (DEGs)

**Fig 1:**
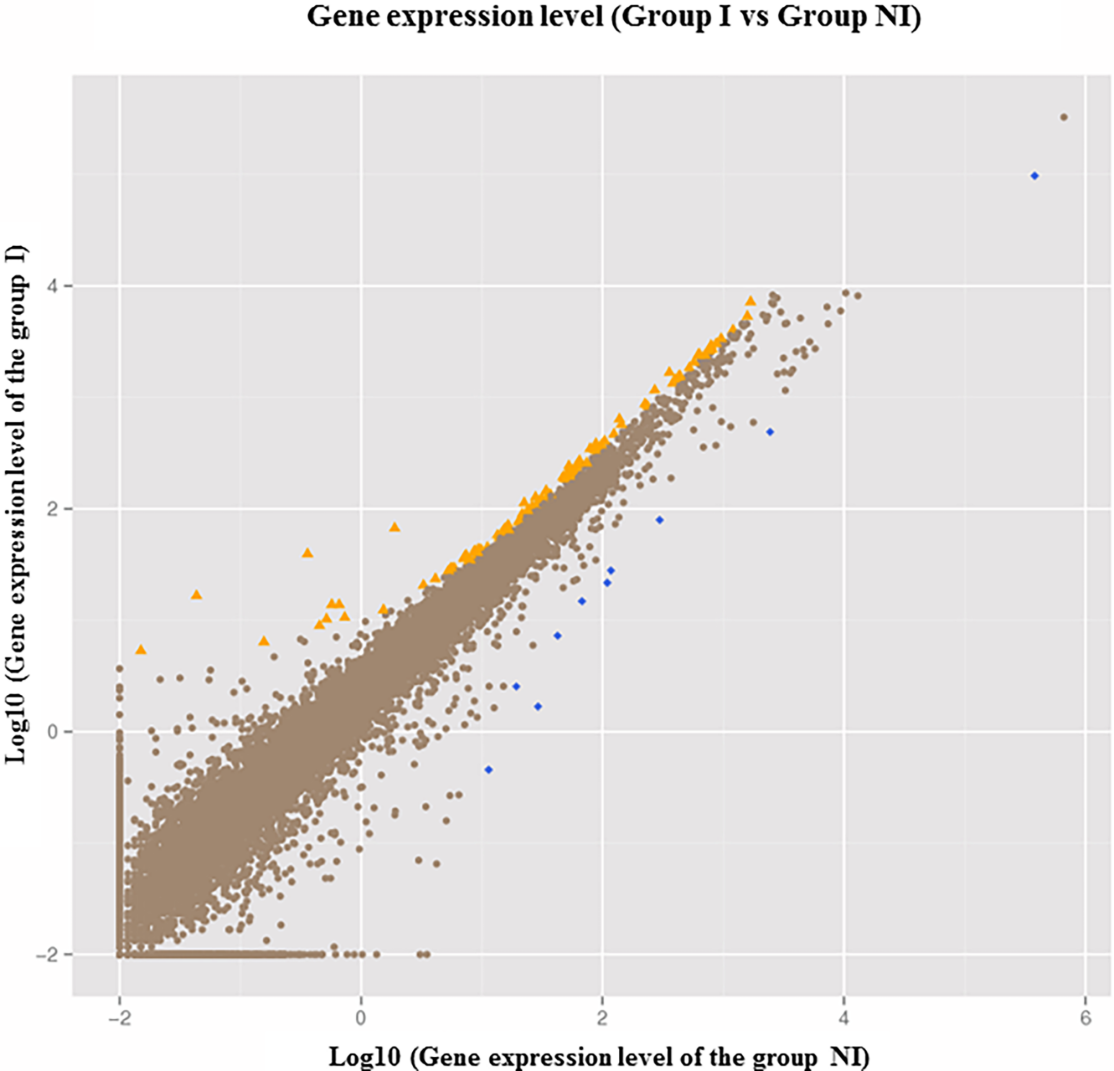
Comparison of gene expression levels between the I and NI groups. Yellow triangles represent up-regulated genes, blue rhombuses indicate down-regulated genes, and brown dots represent genes that did not change significantly. The parameters “Probability> = 0.8” and “abs(log2(Y/X)> = 1)” were used as thresholds to determine the significance of gene expression differences.

In the RNA-seq studies, the expression levels of genes were estimated by counting the reads that mapped to gene exon regions. The read count correlated positively with the length and depth of sequencing. To facilitate transparent comparisons of the expression levels of genes both within and between samples, the parameter reads per kilobase of a gene per million reads (RPKM) was used to normalize the expression levels [21]. Generally, genes with RPKM <1 were set as not expressed, and genes with RPKM>10 were considered to be expressed at high levels. In this study, the RPKM values of most DEGs (90.8%) were less than 3, indicating low expression levels. Among 16,872 assembled unigenes, a total of 110 unigenes were found to be up-regulated, while 10 unigenes were down-regulated in FMDV-infected samples (**Fig 1**). Expression profiles based on the RPKM values of DEGs are summarized in S2 Dataset.

### Functional annotation

**Fig 2:**
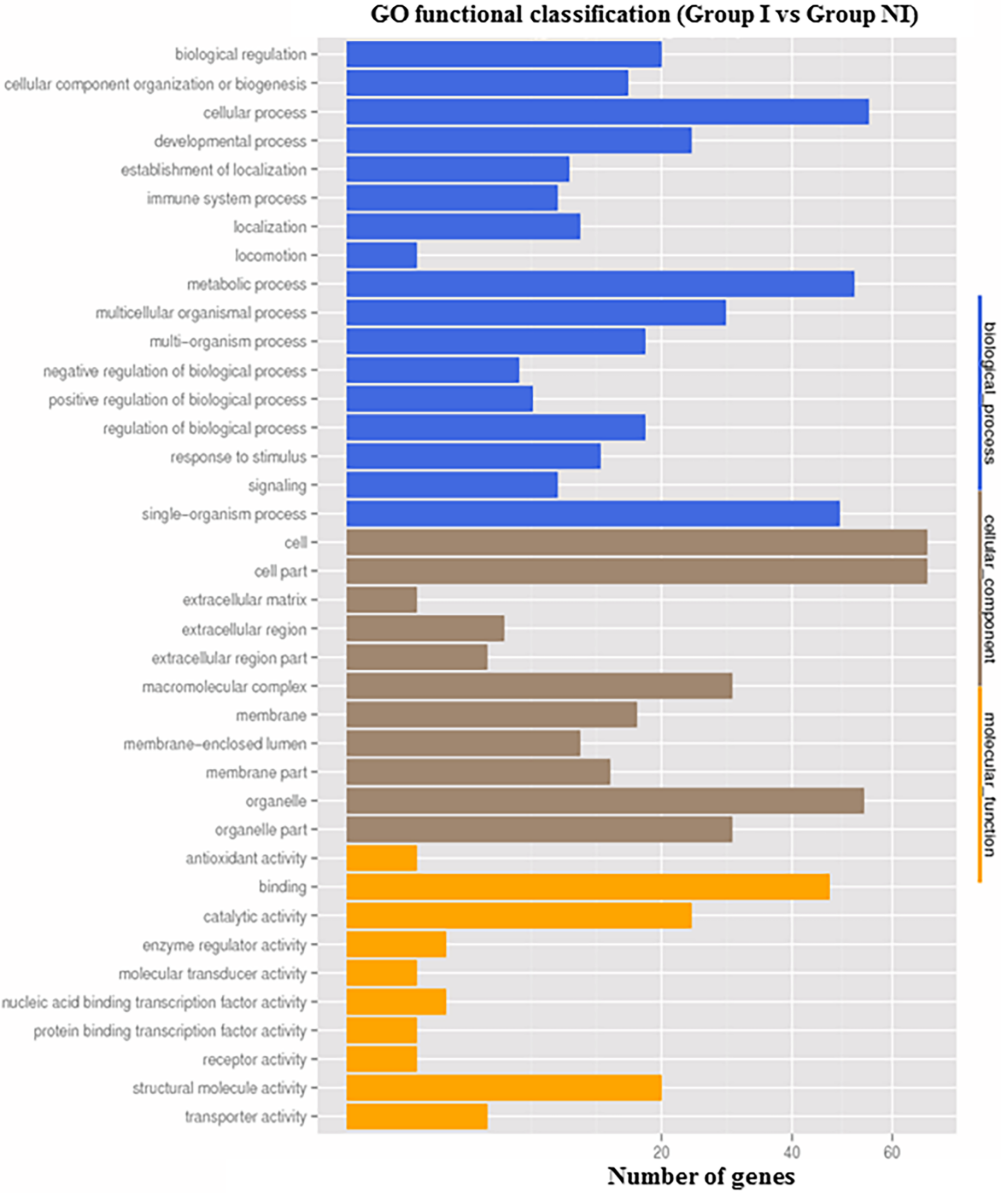
GO annotation of differentially expressed genes. 70 unigenes were annotated for biological process. Most of these genes were assigned into the cellular process subcategory, metabolic process subcategory, and single-organism subcategory. 78 unigenes were annotated for cellular component, and most genes were assigned to the subcategories of cell and cell part. 66 unigenes annotated for molecular function were observed primarily with binding.

GO is an international standardized classification system for categorizing genes and gene products across species. A total of 70 unigenes (64 up-regulated and 6 down-regulated genes) were annotated for biological process. Most of these genes were assigned into the cellular process subcategory (56, 80%), metabolic process subcategory (53, 75.7%) and single-organism subcategory (50, 71.4%). A total of 78 unigenes (72 up-regulated and 6 down-regulated genes) were annotated for cellular component, and most genes were assigned to the subcategories of cell (68, 87.1%), cell part (68, 87.1%), organelle (54, 69.2%) and organelle part (30, 38.5%). The associations of 66 unigenes annotated for molecular function were observed primarily with binding (47, 71.2%), catalytic activity (24, 36.4%) and structural molecule activity (20, 30.3%). Notably, within the biological process category, 8 (11.4%) and 12 (17.1%) unigenes were grouped into the subcategories of immune system process and response to stimulus process, respectively (**Fig 2**).

**Fig 3:**
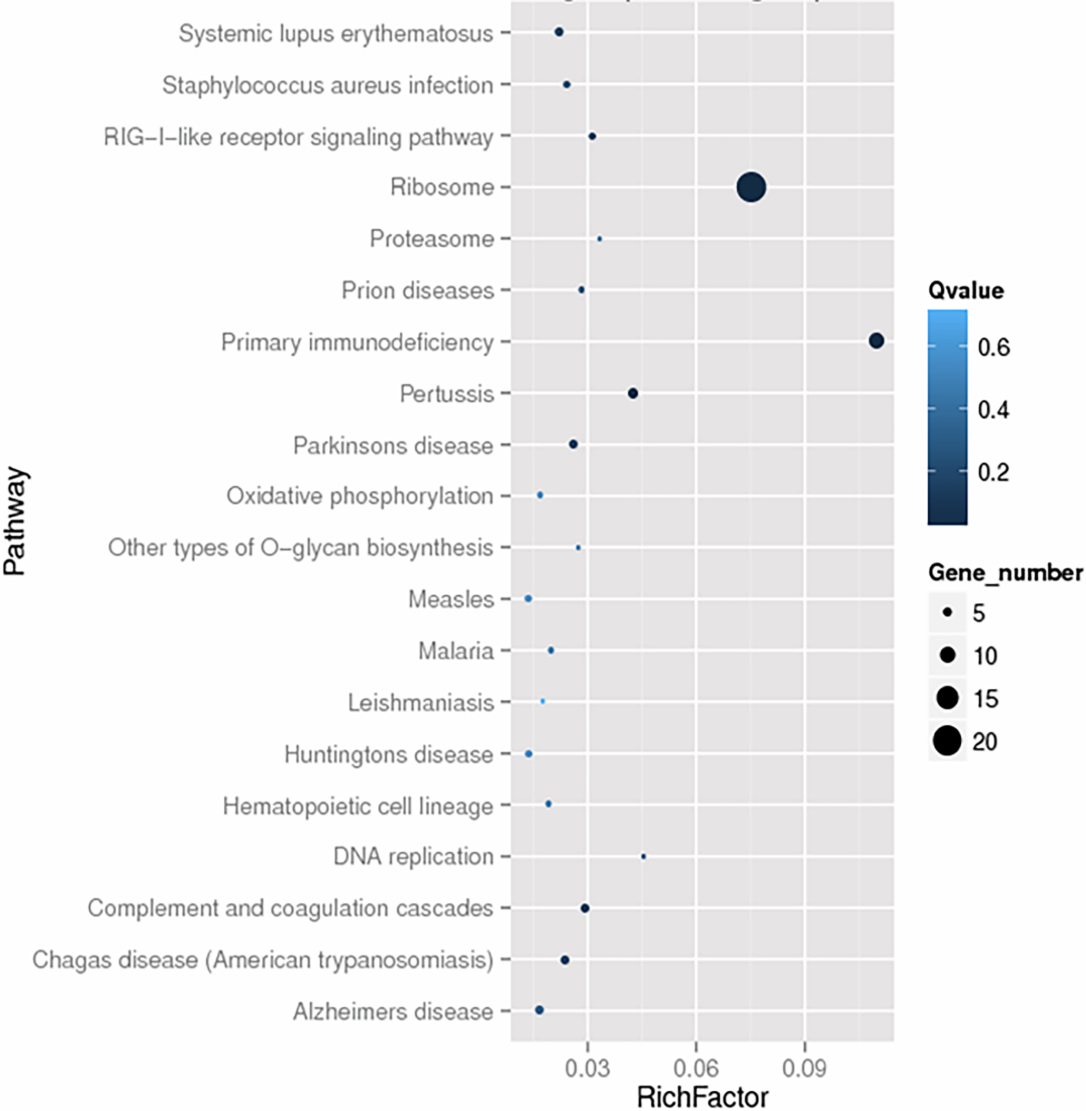
KEGG annotation of differentially expressed genes. KEGG annotation showed that many unigenes are assigned to the ribosome signaling pathway and immune-related pathways.

KEGG is a database resource that is widely used for the integration and interpretation of large-scale datasets generated by high-throughput sequencing technologies. In the present study, the KOBAS software was implemented to test the statistical enrichment of unigenes in the KEGG pathway. The KEGG analysis showed that many unigenes were assigned to several host-virus interaction-related pathways, including the ribosome signaling pathway (18 unigenes), primary immunodeficiency (11 unigenes), the NF-kappa B signaling pathway (5 unigenes) and the RIG-I-like receptor signaling pathway (3 unigenes) (**Fig 3 and S1 Table**).

### Validation of the expression of DEGs by qRT-PCR

**Fig 4:**
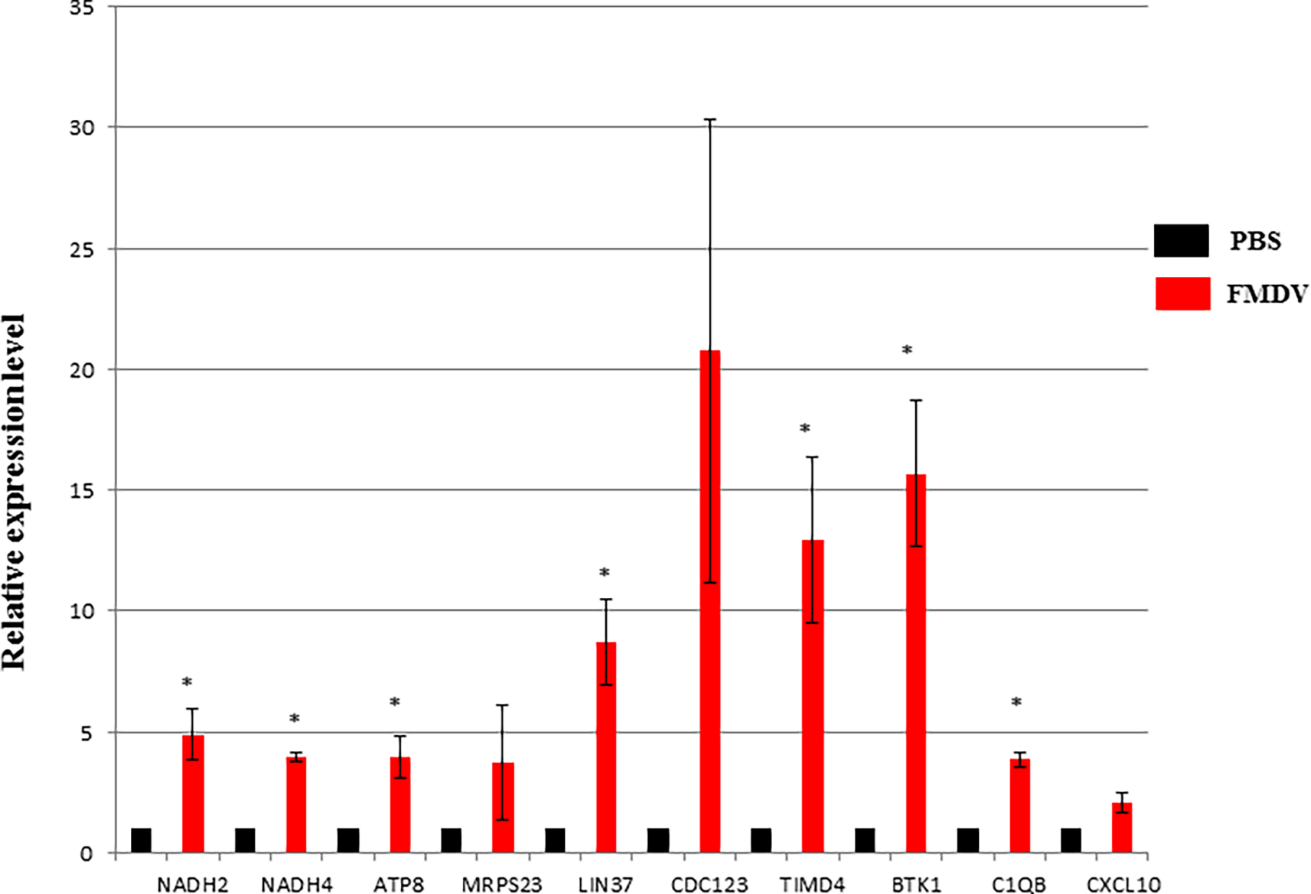
Relative expression levels of 10 randomly selected DEGs. PBS injections were used as controls. β-actin was used as an internal standard. Most of selected genes increased by at least 2-fold compared with the levels in the non-infected group. These experiments were conducted three times. Samples randomly selected for qPCR analysis included NI_1, NI_3, NI_4, I_3, I_4 and I_6.

The results confirmed the up-regulated expression of selected DEGs. The majority of these genes increased by at least 2-fold compared with the levels in the non-infected group. NADH dehydrogenase subunits 2 and 4 and ATP synthase 8 are key components of the system of mitochondrial bioenergetics [22] and increased by at least 4-fold compared with the levels in the control group. MRPS23 is a component of the small mitochondrial ribosome subunit that is involved in combined respiratory chain complex deficiencies [23]. The expression of MRPS23 increased by more than 3-fold compared to that in the non-infected group. LIN37 and CDC123 are involved in the regulation of the cell cycle [24,25], and the expression of both genes increased by over 8-fold. TIMD4, BTK1, C1QB, and CXCL10 are important regulators of innate or adaptive immune responses [26–29], and their expression increased by over 13-, 15-, 4- and 2-fold, respectively (**Fig 4**).

## Discussion

FMD is a highly contagious zoonotic infectious disease that results in huge economic losses to animal husbandry worldwide. Transcriptome analysis is an important method for studying the expression levels and biological function of genes of interest genome-wide. To deepen our understanding of the interactions between FMDV and host cells, transcriptome analysis was used to identify differentially expressed genes genome-wide in porcine whole blood cells infected with FMDV.

After experimentally infecting the pigs with FMDV, we observed several differentially expressed immune response-related genes. The GO and KEGG analysis for functional annotation generated valuable information concerning these DEGs. Out of 99 annotated pathways, 20 were associated with more than 20 unigenes. Among those pathways, the ribosome pathway represented the most abundant group, followed by immune response-related pathways and others. This finding indicated that FMDV influenced protein synthesis in whole blood cells and elicited a broad immune response. Consistent with our findings, Zhao’s study showed that in FMDV-infected peripheral blood mononuclear cells (PBMCs), a number of DEGs were closely associated with protein synthesis and processing pathways [30]. In this study, we used whole blood cells for transcriptome analysis instead of PBMCs. Whole blood cells contain red blood cells, platelets and white blood cells. Due to the lack of nuclei and organelles, red blood cells and platelets do not contain DNA and cannot synthesize any RNA. White blood cells mainly contain two cell populations, PBMCs and polymorphonuclear cells. Polymorphonuclear cells are also important for FMDV infection [31]. It is of significance to include polymorphonuclear cells for transcriptome analysis. Compared with Zhao’s research, we found that some of DEGs identified in our study are different. For instance, according to KEGG annotation, DEGs identified in our study are mainly assigned to the ribosome signaling pathway and immune-related pathways. However, DEGs identified in Zhao’s study are mainly assigned to the protein processing in endoplasmic reticulum pathway and phagosome pathway. The differences in DEGs are mainly due to different cell types and different sampling time.

An increasing number of studies have shown that FMDV infection stimulates diverse signaling pathways, including cell cycle pathways and innate and adaptive host immune cell responses. Interestingly, studies of mitochondrial bioenergetic regulation by FMDV infection have never been reported. It is well demonstrated that viruses can use different strategies to modulate mitochondrial bioenergetics and enhance viral replication [22]. In the present study, we determined that the expression of many genes coding for proteins strongly associated with mitochondrial ATP synthesis increased in the FMDV-infected whole blood cells, indicating that FMDV may be involved in the regulation of mitochondrial bioenergetics in whole blood cells. This information could broaden our targets for FMDV therapy.

## Supporting information

S1 Fig IgG antibody detection in sera detected by liquid-phase blocking ELISA. Antibody titers are expressed as log10 of the reciprocal of the serum dilution giving 50% inhibition of the median absorbance recorded in the antigen control wells.
(TIF)

S2 Fig Amplification of VP1 gene of FMDV by RT-PCR. Total RNA was isolated from vesicle tissues on feet collected from the infection group. Lane1-6: the infected group members I_1 to I_6); Lane 7: positive control; Lane 8: negative control; M: 2000bp DNA marker.
(TIF)

S1 Dataset Quality of clean reads. (XLSX)

S2 Dataset RPKM values of DEGs. (XLSX)

S1 Table Putative different expressed genes involved in immune related pathways or biological activities. (DOCX)
