# Supplementary figures and images for: Gene expression analysis of porcine whole blood cells infected with foot-and-mouth disease virus using high-throughput sequencing technology
Source: PLoS One. 2018 Jul 6;13(7):e0200081. doi: 10.1371/journal.pone.0200081 (PMC6034850; doi:10.1371/journal.pone.0200081)

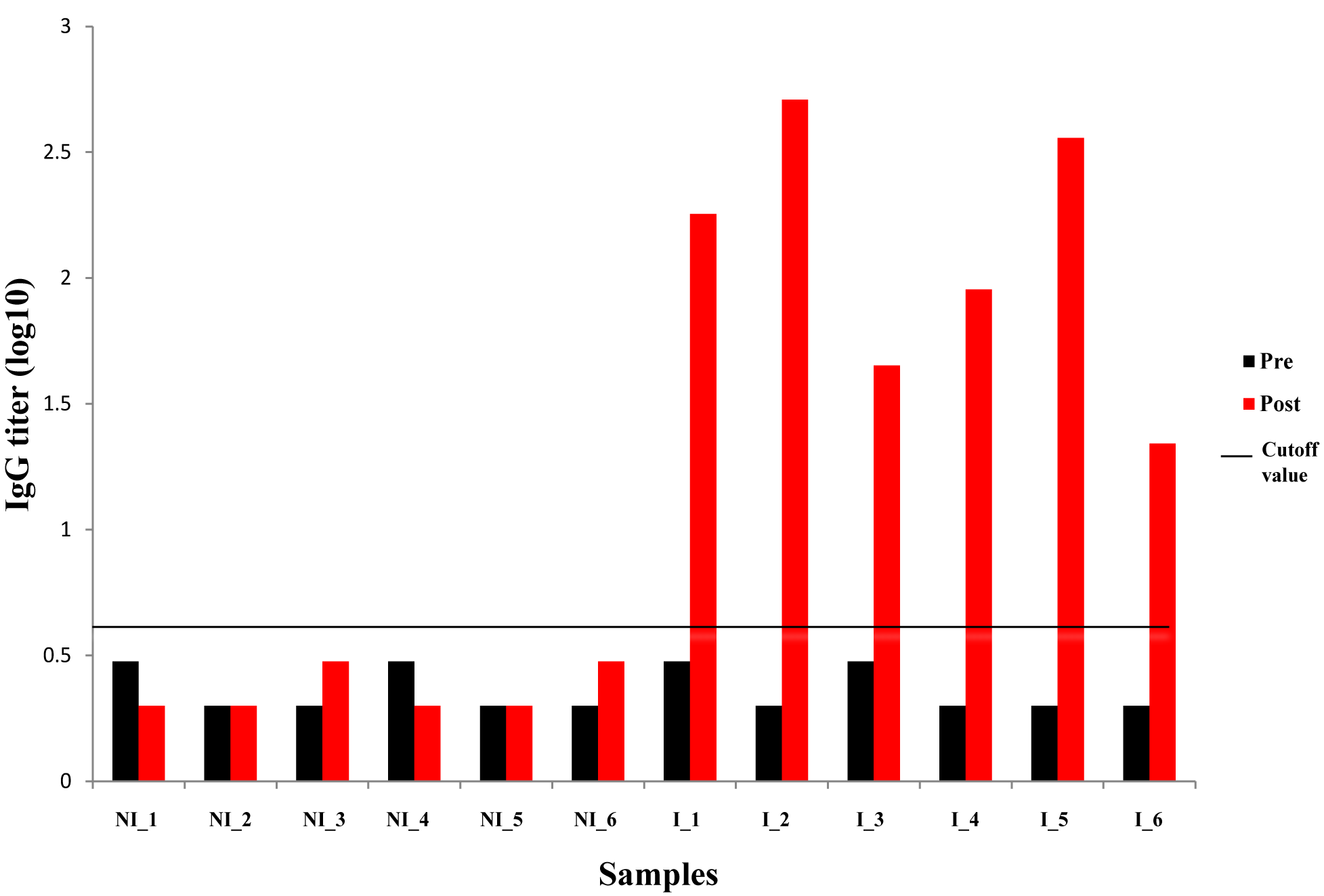

Supplement: S1 Fig — Antibody titers are expressed as log10 of the reciprocal of the serum dilution giving 50% inhibition of the median absorbance recorded in the antigen control wells. (TIF) [file pone.0200081.s001.tif]

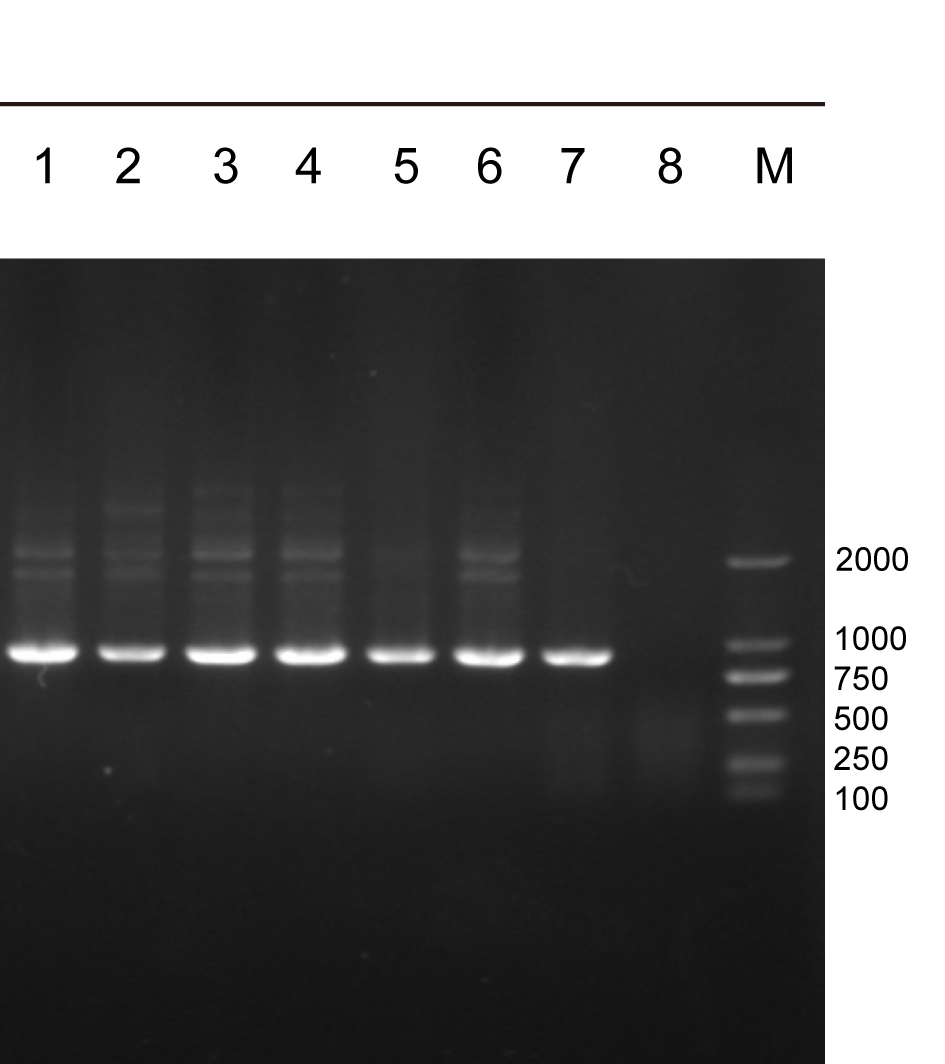

Supplement: S2 Fig — Total RNA was isolated from vesicle tissues on feet collected from the infection group. Lane1-6: the infected group members I_1 to I_6); Lane 7: positive control; Lane 8: negative control; M: 2000bp DNA marker. (TIF) [file pone.0200081.s002.tif]
