# Supplementary material for: Gene expression analysis of porcine whole blood cells infected with foot-and-mouth disease virus using high-throughput sequencing technology
Source: PLoS One. 2018 Jul 6;13(7):e0200081. doi: 10.1371/journal.pone.0200081 (PMC6034850; doi:10.1371/journal.pone.0200081)
Supplement: S1 Table — (DOCX) [file pone.0200081.s005.docx]

**Putative different expressed genes involved in immune related pathways or biological activities.**

| **Pathways** | **ID** | **DEGs with pathway annotation (101)** |
| --- | --- | --- |
| Primary immunodeficiency | ko05340 | 10 (9.90 %) |
| Metabolic pathways | ko01100 | 7 (6.93 %) |
| NF-kappa B signaling pathway | ko04064 | 5 (4.95 %) |
| RIG-I-like receptor signaling pathway | ko04622 | 3 (2.97 %) |
| Toll-like receptor signaling pathway | ko04620 | 2 (1.98 %) |
| Phagosome | ko04145 | 2 (1.98 %) |
| Cytokine-cytokine receptor interaction | ko04060 | 2 (1.98 %) |
| Calcium signaling pathway | ko04020 | 1 (0.99 %) |
| MAPK signaling pathway | ko04010 | 1 (0.99 %) |
| Endocytosis | ko04144 | 1 (0.99 %) |
| T cell receptor signaling pathway | ko04660 | 1 (0.99 %) |
| Antigen processing and presentation | ko04612 | 1 (0.99 %) |
| NOD-like receptor signaling pathway | ko04621 | 1 (0.99 %) |
